# Mitochondrial genome of the Smoothnose wedgefish *Rhynchobatus laevis* from the Western Indian Ocean

**DOI:** 10.1080/23802359.2020.1765209

**Published:** 2020-05-14

**Authors:** Shaili Johri, Anjani Tiwari, Emma N. Kerr, Elizabeth A. Dinsdale

**Affiliations:** a Hopkins Marine Station, Stanford University, Pacific Grove, CA, USA; b Department of Biochemistry, Maharaja Sayajirao University of Baroda, Baroda, India; c Department of Biology, San Diego State University, San Diego, CA, USA

**Keywords:** wedgefishes, conservation, genomics, MinION, wildlife, molecular taxonomy

## Abstract

We present the first mitogenome sequence of the Smoothnose Wedgefish, *Rhynchobatus laevis* obtained through field sequencing on the MinION handheld sequencer. The mitochondrial genome of *R. laevis* is 16,560 bp in length and consisted of 13 protein-coding genes (PCGs), 22 tRNA genes, 2 rRNA genes, and a non-coding control region (D-loop). GC content was at 40.1%. The control region was 867 bp in length. Whole mitochondrial genome sequence of *R. laevis* will enable improved understanding of distribution, abundance, catch and trade rates of the Critically Endangered species.

The Smoothnose Wedgefish (*Rhynchobatus laevis*) is a Critically Endangered IUCN redlist species with a declining population across its range (Peter Kyne 2018). The species is distributed in the Arabian Sea and Bay of Bengal in the Indian Ocean and off China and Japan in the West Pacific (Peter Kyne 2018). It is caught as targeted or incidental catch and is traded heavily for its meat and fins (Peter Kyne 2018). The Smoothnose wedgefish is often confused with members of the *Rhynchobatus djiddensis* complex (Peter Kyne 2018), making it difficult to determine species distribution, abundance, catch rates and trade of the species. We sequenced the whole mitochondrial genome to enable accurate identification of the species through molecular taxonomy. *R. laevis* specimen was collected from the fish market and landing site in Veraval, Gujarat. (Latitude: 20°54′13.1760″, Longitude: 070°22′21.4608″).

Muscle tissue of a juvenile female was stored in RNA later post collection at the Junagadh Agricultural University (Sample accession # Ver_139_3.18.^2018^) and subsequently used for DNA extraction and sequencing following Johri et al., (2019). Approximately 300 Fast5 sequencing files were converted to FASTQ files using the basecaller Guppy 3.3.1 (Oxford Nanopore Technologies) on a GPU interface. Total of 1,199, 267 sequence reads were obtained with a length range of 500–285,000 bp. Reads were trimmed and mapped using the mitogenome from *Glaucostegus granulatus* as reference (Johri et al. 2020), resulting in a contig of 179 reads. The resulting contig consensus sequence was annotated using orthologous loci in *G. granulatus* (Johri et al. 2020).

To assess the phylogenetic position of *R. laevis*, gene trees were constructed using NADH_2_ genes from five families within the order Rhinopristiformes and one family in the order Torpediniformes as outgroup. The NADH2 region was used as opposed to multiple mitochondrial genes or complete mitochondrial genomes due to the sparsely populated genetic database for the species group. All sequences were aligned using MUSCLE 3.8.31 (Edgar 2004) and phylogenies were inferred in a Bayesian inference framework (Huelsenbeck and Ronquist 2001; Ronquist et al. 2012). Bayesian phylogenies were estimated with MrBayes v3.2.6 (Huelsenbeck and Ronquist 2001; Ronquist et al. 2012) using the GTR substitution model, Gamma rate variations with 4 gamma categories, a chain length of 110, 000, burn-in Length of 100, 000 and subsampling frequency of 200. The MrBayes tree (Figure 1) shows that *R. laevis* resides within the clade representing the family Rhinidae and is most closely related to the *R. laevis* NADH2 reference sequence.

**Figure 1.**
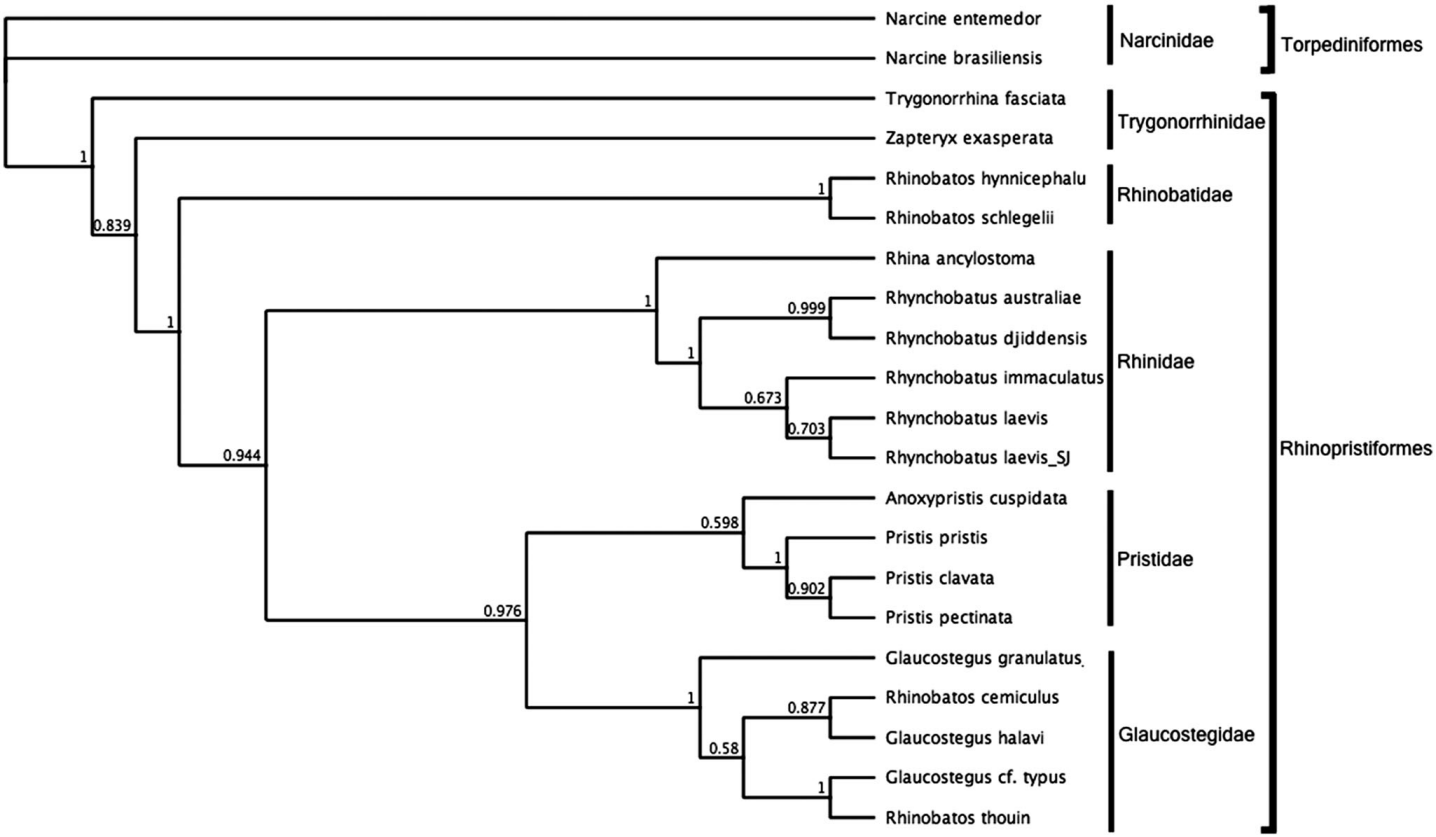
Bayesian estimate of phylogenetic position of *Rhynchobatus laevis* within the order Rhinopristiformes based on the NADH2 mitochondrial region. Members of the order Torpediniformes served as the outgroup. Families are indicated by vertical lines and orders by square brackets. Numbers at nodes are posterior probabilities. GenBank Accession Numbers: *Narcine entemedor* (KM386678.1); *Narcine brasiliensis* (KT119410.1); *Glaucostegus granulatus* (MN783017); *Glaucostegus halavi* _NADH2 (KM396922.1); *Rhinobatos thouin*_NADH2 (JN184264.1); *Rhinobatos cemiculus*_NADH2 (JQ518912.1); Glaucostegus cf. typus_NADH2 (JQ518907.1); Anoxypristis cuspidata (KP233202.1); *Pristis pristis* (MH005928.1); Pristis clavata (KF381507.1); Pristis pectinata (MF682494.1); *Rhina ancylostoma* (KU721837.1); *Rhynchobatus australiae* (KU746824.1); *Rhynchobatus laevis* (MN988687); *Rhynchobatus djiddensis* (JN184077.1); *Rhynchobatus laevis*_NADH2 (JQ519024.1); *Rhinobatos schlegelii* (KJ140136.1); *Rhinobatos hynnicephalus* (KF534708.1); *Zapteryx exasperate* (KM370325.1) and *Trygonorrhina fasciata* (JN184081.1).

The mitochondrial genome of *R. laevis* (GenBank: MN988687) was 16,560 bp in length and consisted of 13 protein-coding genes (PCGs), 22 tRNA genes, 2 rRNA genes, and a non-coding control region (D-loop). GC content was at 40.1%. All PCGs started with ATG and some PCGs ended with an incomplete stop codon. The control region was 867 bp in length. Whole mitochondrial genome sequence of *R. laevis* will enable improved understanding of species distribution, population abundance, catch and trade rates of the species imminently.

## Data Availability

Data that support the findings of this study are openly available in Genbank with reference accession number MN988687.1 at DOI: https://www.ncbi.nlm.nih.gov/nuccore/MN988687.1.
